# Screening for Active Small Molecules in Mitochondrial Complex I Deficient Patient's Fibroblasts, Reveals AICAR as the Most Beneficial Compound

**DOI:** 10.1371/journal.pone.0026883

**Published:** 2011-10-26

**Authors:** Anna Golubitzky, Phyllis Dan, Sarah Weissman, Gabriela Link, Jakob D. Wikstrom, Ann Saada

**Affiliations:** 1 Monique and Jacques Roboh Department of Genetic Research, Department of Genetics and Metabolic Diseases, Hadassah, Hebrew University Medical Center, Jerusalem, Israel; 2 Department of Endocrinology and Metabolism, Hadassah, Hebrew University Medical Center, Jerusalem, Israel; Boston University, United States of America

## Abstract

Congenital deficiency of the mitochondrial respiratory chain complex I (CI) is a common defect of oxidative phosphorylation (OXPHOS). Despite major advances in the biochemical and molecular diagnostics and the deciphering of CI structure, function assembly and pathomechanism, there is currently no satisfactory cure for patients with mitochondrial complex I defects. Small molecules provide one feasible therapeutic option, however their use has not been systematically evaluated using a standardized experimental system. In order to evaluate potentially therapeutic compounds, we set up a relatively simple system measuring different parameters using only a small amount of patient's fibroblasts, in glucose free medium, where growth is highly OXPOS dependent. Ten different compounds were screened using fibroblasts derived from seven CI patients, harboring different mutations.

5-Aminoimidazole-4-carboxamide ribotide (AICAR) was found to be the most beneficial compound improving growth and ATP content while decreasing ROS production. AICAR also increased mitochondrial biogenesis without altering mitochondrial membrane potential (Δψ). Fluorescence microscopy data supported increased mitochondrial biogenesis and activation of the AMP activated protein kinase (AMPK). Other compounds such as; bezafibrate and oltipraz were rated as favorable while polyphenolic phytochemicals (resverastrol, grape seed extract, genistein and epigallocatechin gallate) were found not significant or detrimental. Although the results have to be verified by more thorough investigation of additional OXPHOS parameters, preliminary rapid screening of potential therapeutic compounds in individual patient's fibroblasts could direct and advance personalized medical treatment.

## Introduction

The congenital disorders of mitochondrial oxidative phosphorylation (OXPHOS) are common inborn errors of metabolism, with an incidence of 1:5000–8000 live births [1], [2]. Among these, deficiency of mitochondrial respiratory chain complex I (NADH CoQ oxidoreductase, EC 1.6.5.3) is the most common and accounts for one-third of all patients referred for OXPHOS evaluation [3].

Complex I (CI), is the first complex of the mitochondrial respiratory chain. It is a large multimeric complex composed of 45 structural subunits; seven are encoded by the mitochondrial DNA (mtDNA) while 38 structural subunits and a number of CI assembly factors are all nuclear encoded [4]. Some of the subunits are post transcriptionally modified by phosphorylation, acetylation or glutathionylation [5]–[7].

Disease causing mutations have been identified in all mtDNA encoded subunits as well as in a number of the nuclear encoded complex I subunits and assembly factors [8], [9].

The clinical phenotype of complex I deficiency is varied and includes severe neonatal lactic acidosis, Leigh disease, cardiomyopathy-encephalopathy, hepatopathy-tubulopathy, leukodystrophy with macrocephaly optic atrophy, cerebellar ataxia, retinitis pigmentosa and growth retardation [10]_._ The extensive damage observed in patients with complex I deficiency is most probably due to energy depletion and to over- production of reactive oxygen species (ROS) with subsequent initiation of the apoptotic cascade [11]–[13].

Despite major advances in the biochemical and molecular diagnostics and the deciphering of the CI structure, function, assembly and pathomechanism, there is currently no satisfactory cure for patients with mitochondrial complex I defects. Small molecules provide one feasible therapeutic option, however their use has not been systematically evaluated using a standardized experimental system, and treatment has been based on “trial and error” [14]–[16]. Vitamins (vitamin K, riboflavin, B1,B2), cofactors (CoQ, carnitine, creatine), and ROS scavengers (vitamin E, CoQ_10_), have all been administered to improve OXPHOS, by providing alternate substrates, removing lactate accumulation (by dichloroacetate) and ameliorating oxidative damage (reviewed by Dimauro and Mancuso) [15]–[16]. The favorable effect of Coenzyme Q_10_ supplementation for CoQ deficiency is undisputable however the efficacy of riboflavin has been demonstrated only in a few cases of complex I deficiency [11], [17]. For other compounds, results have been equivocal or were reported anecdotally. In recent years, a large number of compounds with therapeutic potential have been described. These include polyphenolic phytochemicals such as resveratrol, grape seed extract, green tea extract and genistein. Resveratrol is a natural phytoalexin found in a wide variety of plant species, including grapes. Among its numerous properties, resveratrol has been reported to have anti-oxidant activities and to activate the genetic expression of key genes in energy metabolism such as peroxisome proliferator-activated receptor gamma coactivator 1 alpha (PGC1α). Resveratrol and grape seed extract (a proanthocyanidin) were demonstrated to have beneficial effects on mitochondrial function in several experimental models [18]–[20]. Green tea polyphenols attenuated mitochondrial dysfunction in glucose deprived glial cell cultures [21]_._ Genistein is a soy derived isoflavone which has been evaluated in substrate reduction therapy for mucopolysaccharidoses and was also reported to induce mitochondrial biogenesis [22]–[23].

In addition to polyphenols, other substances such as compounds enhancing energy metabolism, antioxidants and chemical chaperones are potentially beneficial.

Representative of this type are 5-Aminoimidazole-4-carboxamide ribotide (AICAR), oltipraz, bezafibrate and sodium phenylbutyrate.

AICAR is a pharmacological activator of AMP activated protein kinase (AMPK).This heterotrimeric protein complex plays a key role in the regulation of energy homeostasis. The kinase is activated by an elevated AMP/ATP ratio caused by cellular and environmental stress, such as heat shock, hypoxia and ischemia. AMPK regulates energy expenditure by modulating NADH^+^ dependent-type III deacetylase SIRT1, resulting in the deacetylation of downstream targets including PGC1α forkhead box O1 and 3 transcription factors [24]. Notably, Thr172 phosphorylation on the AMPK protein is a prerequisite for its activation [25]. Oltipraz is a 1,2-dithiole-3-thione compound with antioxidant properties. Oltipraz has also been demonstrated to reduce apoptosis in cells with chemically inhibited CI by exerting its cytoprotective effect though AMPK [26]–[27]. Bezafibrate is an agonist of peroxisome proliferator-activated receptors (PPARs) stimulating oxidative metabolism and has a documented positive effect on mitochondria. On the other hand, fenofibrate was reported to have a negative effect on CI [16], [28]–[29]. Sodium phenyl butyrate is a, histone deacetylase (HDAC) inhibitor, affecting protein phosphorylation and relief of endoplasmic reticulum stress. Although the mechanism of action of this compound is poorly defined, it has been found to be beneficial in a number of diseases including cancer, neurodegenerative diseases and metabolic diseases [30]–[33]. All of the above mentioned compounds have been documented to exert positive effects, however to our knowledge, they have not been systematically screened in OXPHOS deficient patient's cells together in the same system.

The objectives of this research were two-fold; to develop an *in vitro* system for the rapid screening of multiple compounds using a small amount of fibroblasts from individual patients and to identify compounds with a therapeutic potential for mitochondrial complex I deficiency.

## Materials and Methods

### Subjects

Fibroblasts previously derived (with informed consent, approved by the IRB), from six patients with mitochondrial respiratory chain complex I (CI) deficiency, were included in the study. Most of these patients have been previously described and all harbored known mutations in different nuclear encoded complex I subunits: NDUFS2[34], [8]. NDUFS4 or assembly factors; C6ORF66 (NDUFAF4)[35], C20ORF7[36], FOXRED1 [37], [8] B17.2.L (NDUFA12L)[38]. Their clinical and biochemical data are briefly summarized in Table 1.

**Table 1:** Patient data.

patient/mutated gene	C20ORF7	NDUFS2	NDUFS4	C6ORF66 (NDUFAF4)	FOXRED1	B17.2L (NDUFA12L)
age of onset	1 year	6 months	3 months	birth	4 months	8 months
clinical details	Leigh's syndrome, mild lactic acidosis, died at 6years	optic atrophy, hypo dense basal ganglia, cardio-myopathy, severe lactic acidosis, died at 2years	lactic acidosis, seizures	encephalo-myopathy, severe lactic acidosis, died at 2 months	Micro-cephaly, encephalo-pathy, lactic- acidosis	hypotonia, motor delay, horizontal nystagmus, bilateral optic atrophy, abnormal MRI, mild lactic acidosis
residual muscle enzymatic activities	CI 54% CIV 38% CII,III within normal range	CI 17% C II-V within normal range	CI 25% C II-V within normal range	CI 14% C II-V within normal range	CI 7% C II-V within normal range	CI 24% C II-V within normal range
reference	36	34,11	unpublished	35,12	37	37,13

### Tissue cultures

Fibroblasts were maintained in DMEM (Biological Industries, Kibbutz Beit Haemek, Israel) medium containing 4.5 g glucose per liter and supplemented with 10% fetal calf serum, 50 µg/ml uridine, and 110 µg/ml pyruvate (GLU, permissive medium) at 37°C, 5%CO_2_.

For assessment of various compounds, 3×10^3^ cells/100 µl were seeded in triplicate on three identical 96 well microtiter plates. The following day, the medium was removed, the wells were washed once with phosphate buffered saline (PBS) and replaced with 100 µl GLU medium or a restrictive glucose-free DMEM medium (Biological Industries, Kibbutz Beit Haemek, Israel) supplemented with 10% dialyzed fetal calf serum and 5 mM galactose (GAL) with or without additives as follows: 0.5 mM AICAR (Tocris, Bioscience, Bristol UK); 50 µM Genistein (GENI) (Cayman Chemicals Ann Arbor MI USA; 100 µM, Bezafibrate (BEZA); 10 µM Oltipraz (OLTIP); 5uM Resveratrol (RSV); 10 uM Epigallocatechin gallate (EGCG)(Sigma-Aldrich, Steinheim, Germany) or grape seed extract (GSE) (Ttianjin Jianfeng Natural Products, China). 72h post-treatment the tissue cultures were analyzed for growth, reactive oxygen species (ROS) and ATP. (Biological Industries, Kibbutz Beit Haemek, Israel).

### Assays

Cell growth was measured by a colorimetric method based on the staining of basophilic cellular compounds (mainly nucleic acids, independent on redox status) with methylene blue at A_620_nm, as modified by Jones et al [39]. For the evaluation methylene blue assay (MB) control cells were serially diluted in GLU medium, seeded in six wells. After 48h triplicate wells were measured by MB and triplicate wells were subjected to viable count by trypan blue. For evaluation of time course, 3×10^3^ control cell and cells from a patient were seeded in GLU medium. The following day the medium was replaced either with fresh GLU or GAL. The amount of cells was quantified by MB after 24 h, 48 h, 72 h and 144 h (medium was replaced with fresh after 72 h).

The intracellular ROS production was measured using 2′,7′-dichlorodihydrofluorescein diacetate (DCF) (Biotium Harvard CA USA)[40]. Briefly, growth medium was removed and replaced by 100ul/well of 10 µM DCF in PBS-Ca^2+^ Mg^2+^ (PBS containing 0.9 mM calcium chloride and 0.5 mM magnesium chloride) and the plates were incubated for 20 min at 37°C, 5%CO_2_. After removal of DCF, the ROS production was monitored for 20 minutes in 100ul PBS-Ca^2+^ Mg^2+^ at λ_ex_ 485 nm and λ_em_ 520 nm.

ATP content was measured by luciferin-luciferase using the ATPlite® luminescence assay system according to the manufacturer's instruction (Perkin Elmer Waltham MA, USA).

Luminescence, fluorescence and absorbance measurements were performed with a Synergy HT microplate reader (Bio-Tek instruments, Vinoosky VT, USA).

Relative fluorescence units (RFU) and relative luminescence units (RLU) were calculated by normalizing to growth as measured by MB in parallel wells for each separate experiment. The mean of all experiments was calculated and compared to that of GAL without additive (containing vehicle only). All experiments were performed in triplicate wells on at least two separate occasions. Statistical significance (p<0.05) was calculated by 2-tailed student's t-test. Data were also visualized using the matrix2png software [41]. http://www.bioinformatics.ubc.ca/matrix2png/.

### Fluorescence microscopy

Fibroblasts, were seeded at 3.5×10^4^ cells/ml in GLU medium. The following day the medium was replaced with GLU, GAL or GAL containing 0.5 mM AICAR for 72hrs. For assessment of mitochondrial content and Δψ, the cells grown in 35 mm glass bottom tissue culture, plates and incubated with 200 nM MitoTracker Green FM (MTG) and 50 nM tetramethylrhodamine ethyl ester (TMRE) (Molecular Probes Eugene, Oregon USA), for 90 and 45 minutes respectively at 37°C, 5%CO_2_ [42]. For phospho-AMPK (pAMPK) immunocytochemistry and Mitotracker stain, cells were grown on fibronectin coated coverslips and incubated with 1.5 µM MitoTracker Red CM-H2XRos (MTR, Molecular Probes Eugene, Oregon USA) for 45minutes at 37°C, 5%CO_2_, chased for 30 minutes with the respective growth medium, fixed with 4% paraformaldehyde in PBS, permeabilized with 0.25% Triton X-100, stained with 1:75 Phospho-AMPKα-(Thr172) primary antibody (Cell Signalling Technology Inc.Denver MA,USA) and subsequently, with Dylight 488 conjugated anti rabbit secondary antibody (Jackson Thermo Scientific, Rockford IL,USA). Cells were visualized by fluorescent confocal microscopy (10×4). Image analysis was done with MetaMorph image analysis software. All micrographs in a series were taken under the same conditions.

### Oxygen consumption

Oxygen consumption rate (OCR) was measured using an XF24 extracellular flux analyzer (Seahorse Biosciences, North Billeric,MA,USA). Fibroblast were seeded at 12- 14×10^3^ cells/well in 300 µl in GLU medium on an XF 24 well plate at 37°C, 5%CO_2_. The following day the medium was replaced with GLU, GAL or GAL containing 0.5 mM AICAR. After 72hrs the growth medium was changed to 500 µl unbuffered DMEM medium with the same constituents as above (GLU, GAL or GAL with AICAR) and incubated at 37°C for 1h for equilibration before the measurements. After 10 minutes of OCR baseline measurements, 50 µl carbonylcyanide-3-chlorophenylhydrazone (CCCP) was injected to reach a working concentration of 20 µM and the maximal OCR was measured. Background OCR was measured after injection of rotenone and antimycin to a final concentration of 5 µM each. After the experiment, cell content was estimated by MB and OCR was calculated as OCR minus background divided by MB.

## Results

**Figure 1:**
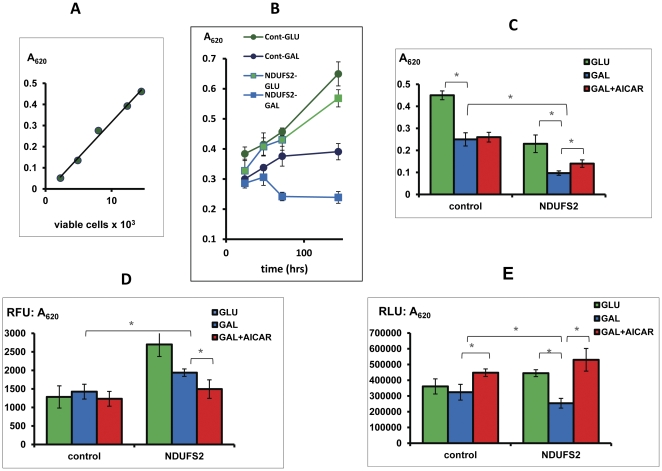
Measurement of growth, ROS and ATP in control and patient fibroblasts. Fibroblast from control and patient (NDUFS2) were grown in microtiter wells in GLU, GAL or GAL supplemented with AICAR. The amount of control cells, measured by methylene blue (MB) at A_620_ was compared to viable count by trypan blue at 72h (A). Growth of control and patient cells was measured at 24,48,72h and 144h by MB at A_620_ (B,C). Growth with AICAR was assessed at 72h (C). ROS measured by DCF at 72h is expressed as relative fluorescent units (RFU) divided by the amount of cells measured by MB at A_620_ (D). ATP was measured at 72h by luciferin-luciferase is expressed as relative luminescence units (RLU) divided by the amount of cells measured by MB at A_620_ (E). Values are presented as mean of triplicates +/- standard deviation. *p<0.05.

All patients presented in infancy, were severely affected and diagnosed with isolated CI deficiency in muscle, with the exception of patient C20ORF7 who had a combined partial deficiency of both CI and CIV. All patients were molecularly defined with mutations either in a nuclear encoded CI subunit or in a CI assembly factor (Table 1). Fibroblasts from these patients were initially assessed for growth, ROS production and ATP content. Preliminary experiments demonstrated that 3×10^3^ cells/well was sufficient to obtain clear readings and reproducible results (Fig. 1A). As it was not possible to measure all parameters in the same well, 3 identical plates treated in parallel were measured in each experiment. Still it was possible to screen ten variables in triplicate wells using less than 3×10^5^ cells (less than a T25 confluent flask). In order to relate values to cell content, we initially evaluated the MB assay and compared those values to values obtained by viable counts. There was a linear relationship between the number of cells and methylene blue absorption (Fig. 1A). As MB staining is much more feasible to perform than viable counting of hundreds of microtiter wells, we therefore continued to evaluate growth by the MB assay. Accordingly, all RFU and RLU values were normalized to MB in order to compare values per cell content. To establish a suitable time frame for the experiments we compared the time course of growth in GLU and GAL. While patient's cell growth was comparable to control cells in GLU media, impaired growth of patient's cells in GAL media was evident after 72h (Fig. 1B). Therefore, 72h was chosen as the optimal time frame for examining compounds.

**Figure 2:**
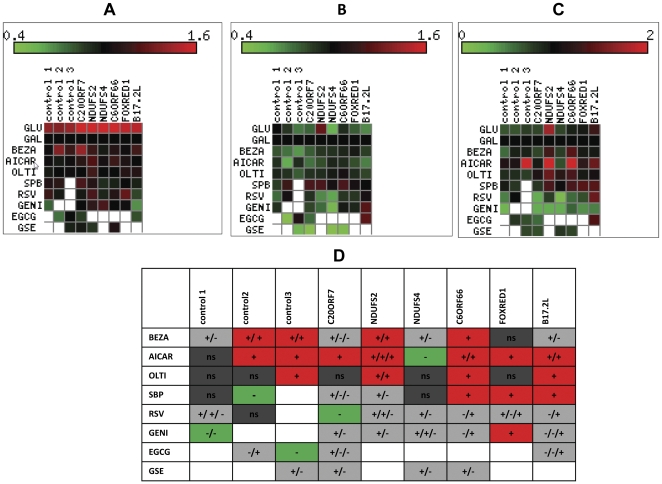
The effect of various compounds on growth, ROS and ATP in control and patient fibroblasts. Fibroblast from three separate controls and seven patients (C20ORF7,NDUFS2,NDUFS4,C6ORF66, FOXRED1 and B17.2L) were grown in microtiter wells in GLU or GAL medium or in GAL medium, supplemented with one of eight various compounds (BEZA,AICAR,OLTI,SBP,RZV,GENI,EGCG,GSI) in GAL for 72hrs. Growth was assessed by MB (A). ROS was measured by DCF and normalized to MB (B). ATP was measured by luciferin-luciferase and normalized to MB (C). Values (A-C) are presented as relative values graphically presented as heatmaps representing the mean of triplicates performed on at least 2 separate occasions. Growth in GAL without additives was set as the value of 1(black). Values < 1 are presented in increasingly green and values >1 in increasingly red. All parameters are summarized in (D) and evaluated as a significantly positive (+), negative (−) or nonsignificant (ns) effect. Increased values for growth and ATP were regarded as positive while negative for increased ROS. Positive only is shaded in red, negative only in green, nonsignificant in dark grey and mixed responses in gray.

A typical experiment with control and patient fibroblasts is depicted in Fig. 1C–D. Since it is difficult to present and summarize a large number (1000<) of measurements, the summary of all data are depicted as a heatmaps in Fig. 2 A–C. (The values on GAL were set at 1 in the middle of the scale (black). Values less than 1 were increasingly green and higher then 1 increasingly red) The growth in GAL medium relative to GLU was slightly decreased in control fibroblasts while growth was markedly decreased in all patients cells (Fig. 2A). These results were anticipated, as GAL medium is devoid of substrates for glycolysis and therefore growth is highly dependent on the oxidation of fatty acids (derived from FCS) by intact OXPHOS for energy production [43]. Intracellular ROS production was generally increased in GAL medium. The increase was evident in one control and four of the patients (Fig. 2B). In patient NDUFS2 the ratio was reversed, presumably because of the severe growth defect in GAL (Fig. 1 B). This is also be the reason for the very low ATP content in GAL medium (Fig. 1C). Notably all control cells had higher ATP content in GAL medium, reflecting the higher efficiency of ATP production by OXPHOS than by glycolysis. This was not the case for four of the six patients and reflects the CI defect. Notably cells with a high ATP content in GAL were derived from controls or patients C2ORF7 and NDUFS4 with a relatively high residual CI activity in muscle (Table 1, Fig. 2C).

Next, the effect of various compounds was examined. The compounds tested were polyphenols, and other compounds with reported effects on ROS production and mitochondrial biogenesis. Untreated cells in the presence of vehicle (DMSO), cells grown in GAL and control cells were included in each experiment. It should be noted that the examination of the effect of different compounds required a prior set of experiments initially based on data available from the literature, in order to optimize conditions with respect to medium and concentration. As these experiments required larger quantities of cells, they were performed in normal cells and in some of the patient's cells. From the preliminary data, we concluded that the effect of additives was best demonstrated under stressful conditions i.e in GAL medium compared to growth with vehicle only in the same medium (Fig. 1B). The effect of each compound on each cell on each of the above parameter is presented in Fig. 2A–C. Many compounds either lacked any effect or had a beneficial effect on growth. For example, bezafibrate increased growth in C20ORF7 approaching that in GLU medium. On the contrary, genistein, EGCG and grape seed extract had a negative effect on growth. Therefore, the investigation of these compounds was not continued in the remaining cells (Fig. 2A). Intracellular ROS production was also favorably affected by many compounds, although mostly by bezafibrate and AICAR. The only compound with an overall negative effect on ROS was sodiumphenylbutyrate (Fig. 2B). AICAR exerted a positive effect on ATP content in four of the six patient cells and one control cell line. Other cells were not affected with the exception of the negative effect on NDUFS4 (Fig. 1C, Fig. 2C).

In order to create a simplified overview, we rated a compound as beneficial when it increased growth, ATP and decreased ROS compared to the values on GAL. The evaluation was designated with a plus sign for each favorable parameter while a negative effect was designated with a minus sign. When no parameter was significantly altered by a compound it was designated non significant (ns). Mixed effects were designated plus/minus (Fig. 2D). To summarize, AICAR was the most favorable compound with positive effects on several parameters in five out of six patient cells. Bezafibrate was also beneficial to two patient' cells but to a lesser extent. Interestingly, Otipraz had a beneficial effect on half the patient's. Although sodium phenylbutyrate slightly increased ROS in some cells, the overall score was positive in fifty percent of the patients (Fig. 2D). No positive but many mixed and some negative effects were observed with resveratrol, EGCG and grapeseed extract. The effect of genistein was unclear since it had a positive effect on only FOXRED1 cells while negatively affected the control.

**Figure 3:**
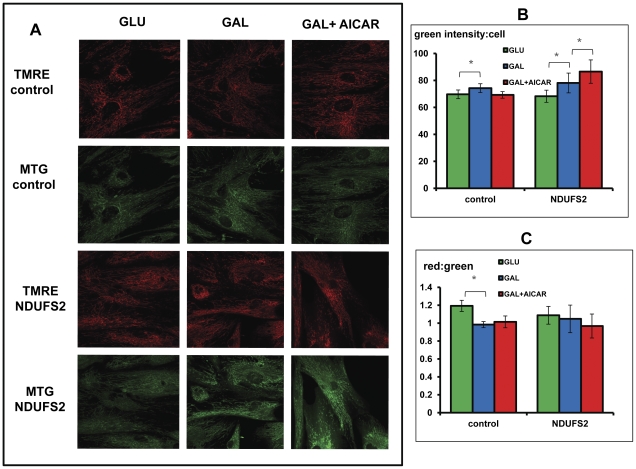
The effect of AICAR on mitochondrial content and Δψ. Control 1 and an NDUFS2 patient's fibroblasts were grown in GLU or GAL medium and in GAL supplemented with 0.5 mM AICAR (GAL+AICAR) for 72 hrs. Cells were then incubated with TMRE (red) and Mitrotracker green (green) and examined by confocal fluorescent microscopy. A: depicts a representative micrograph TMRE stain in red and MTG stain in green. The graphs represent green intensity per cell (B) and red:green ratio (C), +/− standard deviation (*p<0.05). All micrographs were taken under the same conditions.

**Figure 4:**
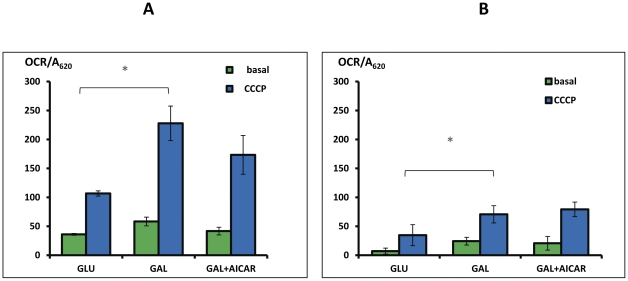
Oxygen consumption. Control 1 (A) and NDUFS2 patient's (B) fibroblasts were grown in GLU or GAL medium and in GAL supplemented with 0.5 mM AICAR (GAL+AICAR) for 72 hrs. Media were changed to unbuffered media and oxygen consumption rates (OCR) were measured by an XF24 instrument. Basal OCR was measured (basal) before the addition of uncoupler and maximal rate (CCCP) after. The results are presented as rates subtracted by non mitochondrial oxygen consumption (in the presence of rotenone and antimycin) and normalized to cell amount measured by methylene blue (MB)at A_620_, +/− standard deviation. *p<0.05.

In order to further investigate the effect of AICAR we conducted an extended study of a representative cell (NDUFS2) to include the evaluation of mitochondrial content and mitochondrial Δψ by fluorescence microscopy and oxygen consumption (Figs. 3 and 4). Mitochondrial content estimated by MTG was found slightly but significantly increased in both control and patients cells in GAL medium. AICAR induced a clear increase in mitochondrial content only in the patient's cells (Fig. 3A and B). The alteration of TMRE stain relative to mitochondrial content in the presence of AICAR was minor and not significant, indicating that Δψ was not substantially affected (Fig. 3A and C). While uncoupled oxygen consumption was increased in GAL medium, AICAR had no significant effect (Fig. 4A and B) although the basal uncoupled OCR was somewhat, not significantly relative to basal OCR in the patient's cells (Fig. 4B).

**Figure 5:**
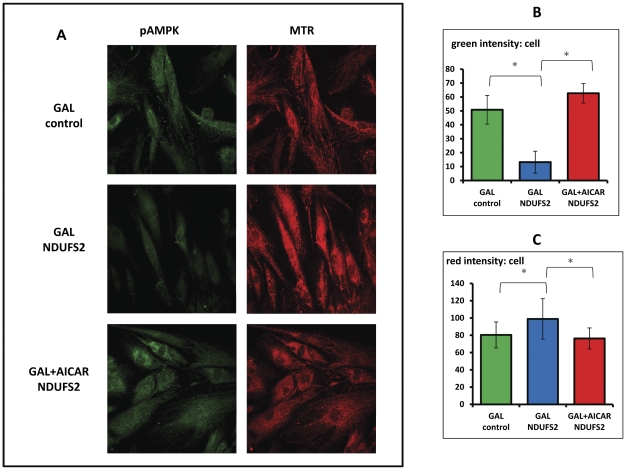
The effect of AICAR on pAMPK and mitotracker red stain in control and patient fibroblasts. Control and patient fibroblasts were grown on coverslips in GLU or GAL medium and in GAL supplemented with 0.5 mM AICAR (GAL+AICAR) for 72 hrs. Cells were than incubated with mitotracker red (MTR), fixed, stained with anti pAMPK antibodies and visualized by fluorescent (pAMPK) secondary antibodies. The coverslips were examined by confocal fluorescent microscopy (10×40). A: depicts a representative migrograph of pAMPK stain in green and MTR stain in red. The graphs represent green intensity per cell (B) and red intensity per cell (C) +/− standard deviation (*p<0.05). All micrographs were taken under the same conditions.

To examine to the downstream effect of AICAR, we performed immunostaining with an antibody towards Thr172 phosphorylated AMPK (pAMPK) while co-staining with MTR (Fig. 5A–C). While clearly present in control cells, pAMPK stain was very weak in NDUFS2 cells grown on GLU (Fig. 5A and B). AICAR supplementation caused a marked and significant increase of pAMPK in the patients cells. Mitotracker stain was increased in patient's cells on GAL but approached control values in the presence of AICAR (Fig. 5A and C).

## Discussion

The search for therapeutic agents for mitochondrial complex I deficiency and OXPHOS defects in general, is seriously hampered by the lack of a standardized model system for evaluating treatment. Many studies focused on small groups of patients simultaneously treated with several agents leading to difficulties in interpreting data and patient responses to therapy *in vivo*. Documented *in vitro* assays usually focus on a specific compound or a specific parameter using a relatively large sample size (11–13,17).

We developed an accessible and relatively simple system in 96 well plates assessing a number of different parameters by the use of one instrument. This enabled us screen multiple compounds on a small amount of fibroblasts simultaneously while measuring a number of different parameters. The small sample size allowed the use of primary cells which is advantageous not only for practical reasons but also because immortalized cells frequently do not retain their original phenotype and thus respond differently than primary cells (personal experience).

The fibroblasts for this study were chosen to represent CI deficiency, the most common OXPHOS defect. The cells were derived from patients with different CI defects, in order to assess individual responses to different compounds. Indeed the responses differed in some instances between the patients. This is exemplified by bezafibrate which was beneficial for NDUFS2 ATP content but not for C20ORF7, emphasizing the need to evaluate compounds on an individual basis. Individual evaluation is especially important when attempting to treat disorders where the mitochondrial function is already *a priori* compromised. The necessity to measure different parameters was evident when observing the effect of the various polyphenolic cytochemicals (resveraterol, EGCG, grape seed extract and geneticin) included in the study. Generally they decreased ROS formation which is advantageous, but concomitantly decreased growth and ATP content. Although some of these results contradict studies reporting positive effects previously mentioned in the introduction, they are actually in accord with other studies reporting that some polyphenols can induce cancer cell death though mitochondrial membrane depolarization and apoptosis [44], [45]. On the other hand, AICAR was found to be the most promising compound with no detected negative effect and an overall positive score in most of the patient's cells. The positive effect on mitochondrial biogenesis was also clearly visible by the MTG stain while the Δψ was not affected. Immunocytochemistry also supported activation of AMPK. Remarkably AICAR has been given intravenously to humans in clinical trials for the treatment of hyperinsulinemia [46]. Recently AICAR was also reported to be favorable in cytochrome c oxidase deficiency [47], [48].

Apparently there is a discrepancy between the mixed effect of resveratrol and the positive effect of AICAR since they both activate the same SIRT1, PGC1α axis pathway [18], [24], [49]. The underlying mechanism for this inconsistency remains unclear and requires further thorough investigation. Nevertheless, we suggest that the positive effects of resveratrol on patients cells might be masked by some additional negative effects. Notably, resveratrol was reported to inhibit the mitochondrial FoF1 ATPsynthase (complex V) and oxygen consumption while depleting ATP content [50], [51]. In fact, resveratrol alone is suggested to act as an anticancer compound by targeting mitochondria through the activation of pro-apoptotic pathways [52]. It is therefore conceivable that resveratrol might exert a negative effect on some parameters on some individual patient's cells with an *a priori* mitochondrial dysfunction. Apart from AICAR, oltipraz and bezafibrate disclosed a general positive effect, but to a lesser extent. Sodium phenylbutyrate increased ATP but also caused a slight increase of ROS and therefore the use of this compound in OXPHOS defects could be questionable.

We detected only a partial correlation between individual responses in fibroblasts and residual enzymatic complex I activity in muscle, genotype or clinical presentation (Table and Fig. 2). Moreover, the clinical correlation between fibroblasts responses and patients response to treatment has only been proved in a few instances and further correlation studies are warranted [11], [17]. Nevertheless patient's fibroblasts provide an accessible tissue for testing individual responses to additives and drugs [48].

Taken together, we present an accessible and relative simple system using a small amount of patient's fibroblasts for screening potential treatments in OXPHOS defects. This enables the screening to be performed on an individual basis while measuring a number of different parameters which are not limited to the measurement of a specific respiratory chain complex. Consequently the system has a wide applicability, and could be used for other defined and undefined OXPHOS defects. The authors are aware that this system is suitable for preliminary screening only, and that the results will have to be verified by precise investigation of additional parameters and mechanisms by other methods measuring OXPHOS by enzymatic assays and expression analysis on the protein and mRNA levels. Nonetheless these assays are more complex and require a larger number of cells making them much less applicable for screening purposes. We propose that rapid preliminary screening of potential therapeutic compounds in individual patient's fibroblasts could direct and advance personalized medical treatment.
